# Integrating moving average control procedures into the risk-based quality control plan in small-volume medical laboratories

**DOI:** 10.11613/BM.2022.020711

**Published:** 2022-06-15

**Authors:** Vera Lukić, Svetlana Ignjatović

**Affiliations:** 1 Department of Laboratory Diagnostics, Railway Healthcare Institute, Belgrade, Serbia; 2 Department of Medical Biochemistry, University of Belgrade, Faculty of Pharmacy, Belgrade, Serbia; 3 Center for Medical Biochemistry, Clinical Center of Serbia, Belgrade, Serbia

**Keywords:** risk-based quality control plan, QC frequency, run size, patient-risk sigma metrics, patient-based real-time quality control

## Abstract

**Introduction:**

The modern approach to quality control (QC) in medical laboratories implies the development of a risk-based control plan. This paper aims to develop a risk-based QC plan for a laboratory with a small daily testing volume and to integrate the already optimized moving average (MA) control procedures into this plan.

**Materials and methods:**

A multistage bracketed QC plan for ten clinical chemistry analytes was made using a Westgard QC frequency calculator. Previously, MA procedures were optimized by the bias detection simulation method.

**Results:**

Aspartate aminotransferase, HDL-cholesterol and potassium had patient-risk sigma metrics greater than 6, albumin and cholesterol greater than 5, creatinine, chlorides, calcium and total proteins between 4 and 5, and sodium less than 4. Based on the calculated run sizes and characteristics of optimized MA procedures, for 6 tests, it was possible to replace the monitoring QC procedure with an MA procedure. For the remaining 4 tests, it was necessary to keep the monitoring QC procedure and introduce MA control for added security.

**Conclusion:**

This study showed that even in a laboratory with a small volume of daily testing, it is possible to make a risk-based QC plan and integrate MA control procedures into that plan.

## Introduction

Quality control (QC) is one of the basic principles behind the analytical work of medical laboratories and guarantees the reliability of laboratory results. Traditional quality control of analytical work is performed by testing commercially available control materials at certain time intervals. Statistical rules are then applied to the obtained results of control measurements to check their acceptability (*1*). Westgard’s control rules are the most commonly used, introduced 50 years ago. This traditional concept of statistical QC has been well developed. Nevertheless, there are significant differences in how laboratories conduct it, especially in terms of the frequency of control measurements and the number of control rules they apply (*2*). The main disadvantages of traditional QC are: intermittency, the problem of commutability, and the costs of materials and labour (*3*). Due to the intermittency of this type of control, there is a risk that the analytical bias that occurs between two control measurements will remain undetected, causing erroneous patient results to be issued (*4*). Therefore, determining the frequency of control measurements is of crucial importance. The 2016 C24-Ed4 guideline of the Clinical and Laboratory Standards Institute (CLSI) – Statistical Quality Control for Quantitative Measurement Procedures: Principles and Definitions – recommends developing a laboratory QC plan based on risk (*5*). This refers to a risk for the patient due to clinical decisions potentially based on a laboratory result that contains an error. The concept of bracketed control has thus been introduced. At certain intervals, control samples are measured and interpreted together with patient samples, suggesting that if controls are acceptable, the patient results obtained between the two controls are also acceptable (*6*). The frequency of control measurements in a risk-based plan is defined based on the Max E(N_uf_) parameter, which represents the maximum expected number of unreliable final patient results issued during the presence of an undetected error condition between two control measurements (*7*). The calculation of this parameter is very complex and therefore impractical for routine use in laboratories (*8*). The practical information that laboratories actually need to establish QC frequency is the run size, *i.e.*, the number of patient samples between two control measurements (*9*). The calculation has been simplified by designing a run size nomogram between two control measurements (*10*) and recently further facilitated due to the availability of an online run size calculator on the Westgard website (*9*). In order to use this nomogram, as well as the calculator, it is necessary to know the Sigma-metric of a laboratory test.

Sigma-metric is a measure of quality that quantifies the characteristics of the measurement process as the number of defects per million products (*11*). Tests whose Sigma-metric has low value require control strategies that are complex and expensive in terms of frequency, number of control samples, and number of control rules that must be applied. For these tests, the introduction of patient-based real-time quality control (PBRTQC) procedures into a routine quality control plan may be considered (*6*). PBRTQC is a concept of quality control that involves using patient results generated by a laboratory on a daily basis for control purposes (*12*). Unlike the traditional one, this type of control is continuous, free from commutability issues, and requires no costs for control material (*13*, *14*). One of the ways to use patient results for analytical QC is the moving average (MA) (*15*). Moving average involves calculating an average value from the obtained set of patient results and further using that value for control purposes. The MA value is recalculated each time a new result is received from the analyser, *i.e.*, the data are continuously updated and evaluated as patient samples are analysed. Moving average procedures are specific to each test and laboratory and, therefore, cannot be generalized or taken from another source but require individual selection, optimization and validation (*16*). The complexity of determination is the main reason PBRTQC has not become widespread in laboratories, despite being known for decades (*15*). However, thanks to the availability of software applications that perform all the necessary calculations, in recent years, interest in this form of QC has resurfaced (*17*). In the case of laboratories with a small volume of daily testing, both the issue of developing a risk-based QC plan and the implementation of MA control procedures have been insufficiently researched.

This paper aims to develop a risk-based QC plan in a laboratory with a small daily volume of testing and to integrate the MA control procedures the laboratory already has into this plan.

## Materials and methods

### Materials

The study was performed as a retrospective analysis of data from the laboratory information system (LIS) and the external and internal quality control at the Department of laboratory diagnostics, Railway Healthcare Institute, for the period July 2019 to June 2020. The laboratory provides services to a general adult population at the primary level of healthcare, with an average annual number of about 400,000 tests. The following 10 analytes were included in the study: albumin, aspartate aminotransferase (AST), creatinine, calcium, chloride, cholesterol, HDL (high-density lipoprotein)-cholesterol, potassium, sodium, and total proteins. These analytes were chosen because the laboratory already has optimized MA procedures implemented in the LIS (Next lab, BitImpex, Belgrade, Serbia). All tests were performed on the Architect c16000 clinical chemistry analyser (Abbott, Abbott Park, USA) with the original reagents. Consent for the use of data from the LIS in this study was obtained from the Ethical Committee of the Railway Healthcare Institute.

### Methods

#### Calculation of sigma metrics

For all 10 tested analytes, sigma metrics were calculated according to the formula: Sigma = (TEa - Bias) / CV, where all values in the formula are expressed in %.

The total allowable error (TEa) value was taken from the Clinical Laboratory Improvement Amendments (CLIA) data (*18*). For tests for which CLIA doesn’t give a percentage but an absolute value of TEa (calcium, potassium, and sodium), the percentage values were calculated related to the target value of each level of control material.

The coefficient of variation (CV) was calculated based on internal QC (IQC) data from the clinical chemistry analyser for the period of 6 consecutive months. One lot of commercially available control material (Multichem S, Technopath, USA) with 3 levels of value was used.

The bias value for each analyte was calculated based on the results of a monthly external QC program the laboratory participates in (EQAS, BioRad, USA). From the external control results for 12 consecutive months, those values were selected that correspond to the target values of the analyte in the levels of the material used for IQC. For each of the three levels of internal control, 3 external control samples were found in corresponding concentrations. For each of these groups of three samples, the arithmetic mean of the bias obtained in external control was calculated, and this value was considered the bias for the corresponding analyte concentration level.

#### Development of a multistage bracketed risk-based QC plan

A multistage bracketed risk-based QC plan was developed for each examined analyte using the Westgard QC Frequency or Run Size Calculator (*19*). The following data must be entered in the calculator for each level of control material (in our case 3): target concentration, precision, bias, and TEa. Target concentrations were taken from the manufacturer’s value list, and precision, bias, and TEa in the manner already explained when calculating sigma metrics. The Patient Risk Factor corresponding to the MaxE (Nuf) value was set in the calculator to 1. This ensured that the number of erroneous patient results would not exceed one if an analytical error occurred between two QC events (*9*). For each control material level, in addition to the calculated sigma metric value, the calculator gives a Patient-risk sigma value. Patient-risk sigma is equal to calculated sigma when this is less than or equal to 6; if the calculated sigma is greater than 6, the patient-risk sigma value is always 6. Besides individual control levels, the calculator also calculates the average patient-risk sigma as the arithmetic mean of patient-risk sigma values for all 3 levels. We further considered this average patient-risk sigma value the sigma value of the test. Based on it, we classified the tests into 3 groups to design a control strategy: high sigma strategy (sigma ≥ 5.0), medium sigma strategy (4.5 ≤ sigma < 5.0) and low sigma strategy (3.5 ≤ sigma < 4.5) (*20*). Finally, the calculator provides control procedures that are suitable candidates for designing a risk-based control strategy, along with the run size of the patients’ results that need to be bracketed by the control.

Candidate statistical quality control (SQC) procedures if using 3 levels of control material are presented in Table 1.

**Table 1:** Candidate SQC procedures and meaning of the control rules

**SQC procedure**	**N**	**Pfr (%)**	**Meaning of the control rule**
			Analytical run is rejected if…
1:3s/ 2of 3:2s/ 3:1s/ 6:X	6	0.07	one QC result exceeds 3 SD limit / or two of three QC results exceed 2 SD limit / or three QC results exceed 1 SD limit/ or six consecutive control measurements fall on one side of the mean
1:2.5s	6	0.06	one QC result exceeds 2.5 SD limit
1:3s/ 2of 3:2s/ 3:1s	3	0.02	one QC result exceeds 3 SD limit / or two of three QC results exceed 2 SD limit / or three QC results exceed 1 SD limit
1: 2.5s	3	0.03	one QC result exceeds 2.5 SD limit
1: 3s	3	0.01	one QC result exceeds 3 SD limit
1: 2s	1	0.05	one QC result exceeds 2 SD limit
1: 2.5s	1	0.01	one QC result exceeds 2.5 SD limit
1: 3s	1	0	one QC result exceeds 3 SD limit
N – number of control measurements *per* QC event. SQC – statistical quality control. Pfr – probability for false rejection. SD - standard deviation. QC - quality control.			

Before including the data from the calculator in the QC plan, we defined the maximum daily number of tests in our laboratory (based on LIS data for the previous 6 months) and the desired reporting interval (size of the series of patient samples after which the results are issued to users). Due to the small daily number of tests in our laboratory (for the tested analytes, this number ranges from 30 to 150 *per* day) and the work organization (one-shift work without urgent service or emergency requests), we chose a reporting interval equal to the daily number of tests. Following guidelines from the literature, we then made a start-up QC plan, which would be implemented at the beginning of each working day before the analysis of patient samples, and a monitoring QC plan, which would be implemented periodically during working day (in our case, at the end of the day) (*6*).

As the start-up plan, we selected the SQC design whose run size, according to the calculator, was equal to or greater than the estimated number of daily tests we perform. The monitoring QC plan was the SQC whose run size, according to the calculator, was equal to or greater than our desired reporting interval and whose Pfr (specified in the Run size calculator) was ≤ 0.05.

#### Selecting the optimal MA procedure

The selection of the optimal MA procedure for each of the 10 examined analytes was performed using the bias detection simulation method introduced by Van Rossum (*16*, *21*). The process of optimization and validation of MA procedures and their implementation in the LIS has been previously described in detail (*22*, *23*). In short, for each MA procedure were selected a calculation formula (simple MA or exponentially weighted MA-EWMA), block size or weighting factor (depending on the formula), inclusion limits (cut-off limits) and control limits. The introduction of a bias of - 50% to + 50%, including a TEa bias, was then simulated by the software. Based on data from the literature, we considered TEa to be clinically significant bias, *i.e.*, the key parameter for optimization was the ability of an MA procedure to detect bias of TEa size within the total number of daily tests performed by the laboratory (*1*, *4*, *13*, *17*). The number of patient results required to detect bias of a certain size was obtained from MA validation graphs (*22*). The median number of results needed to detect a particular bias means that in 50% of cases, the bias will be caught in less than and in 50% of cases more than that number of results (*21*). In the study, we used previously obtained data on the ability of each of these MA procedures to detect clinically significant bias (*23*).

#### Integration of a bracketed SQC plan with MA procedures

Finally, we compared the run sizes provided by the SQC plan with the ability of optimized MA procedures to detect bias equal to TEa within the maximum daily number of tests. Based on these data, we assessed how MA procedures could be added to the bracketed SQC plan.

#### Statistical analysis

All MA calculations and simulations were performed using the dedicated software MA Generator (Huvaros B.V., Bloemendaal, The Netherlands) (*24*).

## Results

Table 2 shows the input data entered into the Run-size calculator for all 3 IQC levels of each of the 10 examined analytes and the sigma metric values provided by the calculator for each of them.

**Table 2:** Sigma metrics for each control level of 10 tested analytes and average sigma metrics for all 3 control levels

	**QC – Level 1**					**QC – Level 2**					**QC – Level 3**					**Average**	
	**TEa** **(%)**	**Bias** **(%)**	**CV** **(%)**	**Sigma** **(%)**	**Risk Sigma** **(%)**	**TEa** **(%)**	**Bias** **(%)**	**CV** **(%)**	**Sigma** **(%)**	**Risk Sigma** **(%)**	**TEa** **(%)**	**Bias** **(%)**	**CV** **(%)**	**Sigma** **(%)**	**Risk Sigma** **(%)**	**Av Sigma** **(%)**	**Av Risk** **Sigma (%)**
Albumin	10.00	2.77	1.60	4.52	4.52	10.00	2.24	0.88	8.82	6.00	10.00	1.91	0.92	8.79	6.00	7.38	5.51
AST	20.00	2.47	2.15	8.15	6.00	20.00	1.14	1.36	13.87	6.00	20.00	0.97	1.15	16.55	6.00	12.86	6.00
Calcium	15.43	2.40	3.01	4.33	4.33	10.55	1.87	2.21	3.93	3.93	8.14	2.39	1.43	4.02	4.02	4.09	4.09
Chloride	5.00	0.58	1.23	3.59	3.59	5.00	0.75	0.91	4.67	4.67	5.00	0.65	0.52	8.37	6.00	5.54	4.75
Cholesterol	10.00	2.50	1.62	4.63	4.63	10.00	2.79	1.35	5.34	5.34	10.00	2.71	1.42	5.13	5.13	5.03	5.03
Creatinine	15.00	2.12	3.13	4.12	4.12	15.00	1.85	2.96	4.44	4.44	15.00	0.97	2.42	5.80	5.80	4.79	4.79
HDL- cholesterol	30.00	2.45	3.11	8.86	6.00	30.00	2.56	3.41	8.05	6.00	30.00	3.68	3.46	7.61	6.00	8.17	6.00
Potassium	18.66	1.38	0.95	18.19	6.00	11.09	1.02	1.01	9.97	6.00	7.54	0.77	0.92	7.36	6.00	11.84	6.00
Sodium	3.22	0.26	0.73	4.05	4.05	2.76	0.40	0.55	4.29	4.29	2.38	0.28	0.63	3.33	3.33	3.89	3.89
Total protein	10.00	1.10	2.46	3.62	3.62	10.00	1.65	2.06	4.05	4.05	10.00	3.31	1.48	4.52	4.52	4.06	4.06
TEa from CLIA, Bias from EQAS, CV from IQC. All sigma values were calculated using a Run-size calculator. Patient-risk factor in all calculations was 1. TEa - total allowable error. CV – coefficient of variation. QC – quality control. IQC – internal quality control. AST – aspartate aminotransferase. HDL-cholesterol – high-density lipoprotein-cholesterol. Av Sigma – average calculated sigma. Av Risk Sigma – average patient-risk sigma.																	

Of the 10 tests examined, albumin, AST, cholesterol, HDL-cholesterol and potassium had a patient-risk sigma greater than 5, chloride and creatinine between 4.5 and 5, calcium and total protein between 4 and 4.5, and the worst was sodium with a calculated sigma of 3.89. Based on that, the tests were divided into 3 groups for designing the QC strategy, which is shown in Table 3.

**Table 3:** Categorization of 10 examined analytes according to patient-risk sigma metric values

**Category for risk-based SQC strategy**	**Sigma value range**	**Analyte**
High sigma	sigma ≥ 5.0	Albumin, AST, cholesterol, HDL-cholesterol, potassium
Moderate sigma	4.5 ≤ sigma < 5.0	Chloride, creatinine
Low sigma	3.5 ≤ sigma < 4.5	Calcium, sodium, total protein
SQC – statistical quality control. AST – aspartate aminotransferase. HDL-cholesterol – high-density lipoprotein-cholesterol.		

Table 4 shows the run sizes calculated by the Run-size calculator for the different control rules. From Table 4, we chose the simplest multi-rule for the start-up QC procedure, which fits all examined analytes and includes 3 measurements (to cover the widest possible range of concentrations through three levels of control material). In our case, it is the 1:3s / 2of 3:2s / 3:1s N3 multi-rule with a false rejection probability of Pfr = 0.02. For the monitoring QC procedure, which brackets the end of a daily series, we selected a single QC rule with one measurement that again fits all examined analytes, and it is 1: 2s N1 with the probability of rejection Pfr = 0.05 (run size for sodium is border adequate). Looking for a run size appropriate for all 10 tests simultaneously, it was not possible to select a rule with a smaller Pfr.

**Table 4:** Run sizes for candidate control procedures for 10 examined analytes

**Analyte** **(Maximal daily number of tests = desired reporting interval)**	**Albumin** **(30)**	**AST** **(150)**	**Calcium** **(30)**	**Chloride** **(50)**	**Cholesterol** **(120)**	**Creatinine** **(120)**	**HDL** **(120)**	**Potassium** **(50)**	**Sodium** **(50)**	**Total protein** **(30)**
**Control procedure**										
1:3s/ 2of3:2s/ 3:1s/ 6:X N6 (p_fr_ 0.07)	1000	1000	842	1000	1000	1000	1000	1000	453	770
1:2.5s N6 (p_fr_ 0.06)	1000	1000	926	1000	1000	1000	1000	1000	522	853
1:3s/ 2of3:2s/ 3:1s N3* (p_fr_ 0.02)	1000	1000	178	1000	1000	1000	1000	1000	100	164
1:2.5s N3 (p_fr_ 0.03)	1000	1000	234	1000	1000	1000	1000	1000	142	218
1:3s N3 (p_fr_ 0.01)	1000	1000	51	284	590	308	1000	1000	30	47
1:2s N1^†^ (p_fr_ 0.05)	1000	1000	70	253	436	269	1000	1000	47	66
1:2.5s N1 (p_fr_ 0.01)	390	1000	25	91	156	96	1000	1000	17	24
1:3s N1 (p_fr_ 0.00)	140	363	9	33	56	35	363	363	6	9
Run size for each candidate control procedure calculated by Run size calculator. *Selected for start-up QC procedure. ^†^Selected for monitor QC procedure. N – number of control measurements *per* QC event. Pfr – probability for false rejection. AST – aspartate aminotransferase; HDL – high-density lipoprotein cholesterol.										

The characteristics of the MA procedures already implemented in our laboratory for the 10 examined analytes are given in Table 5.

**Table 5:** Characteristics of optimized MA procedures for 10 examined analytes

**Analyte**	**Type of MA procedure**	**Bias direction**	**Number of results needed to detect a bias equal to the TEa**		
			Minimum	Median	Maximum
Albumin	Simple MA Block size: 10	negative	6	7	13
		positive	5	7	19
AST	Simple MA Block size: 100	negative	20	55	120
		positive	44	77	118
Calcium	Simple MA Block size: 10	negative	6	8	10
		positive	4	10	16
Chloride	Simple MA Block size: 10	negative	4	7	17
		positive	5	8	10
Cholesterol	Simple MA Block size: 25	negative	14	44	134
		positive	28	65	225
Creatinine	EWMA Weighting factor: 0.1	negative	6	20	44
		positive	27	86	360
HDL-cholesterol	Simple MA Block size: 25	negative	14	18	24
		positive	13	18	65
Potassium	EWMA Weighting factor: 0.1	negative	4	8	13
		positive	5	9	18
Sodium	Simple MA Block size: 25	negative	11	14	18
		positive	4	7	10
Total protein	EWMA Weighting factor: 0.05	negative	8	12	17
		positive	5	9	13
MA – moving average. EWMA – exponentially weighted moving average. TEa – total allowable error. AST – aspartate aminotransferase. HDL-cholesterol – high-density lipoprotein cholesterol.					

The data in Table 4 show that optimized MA procedures will undoubtedly detect a clinically significant bias within the daily number of tests for 8 of the 10 analytes examined.

Considering the data from Tables 3, 4 and 5, we made a multistage risk-based QC plan shown in Table 6.

**Table 6:** Multistage risk-based QC plan

**Analyte**	**Start-up** **QC procedure**	**Monitor** **QC procedure**
Albumin, AST, chloride, cholesterol, HDL-cholesterol, potassium	1:3s/ 2of3:2s/ 3:1s N3	MA
Calcium, creatinine, sodium, total protein	1:3s/ 2of3:2s/ 3:1s N3	1:2s N1 and MA
QC – quality control. AST – aspartate aminotransferase. HDL-cholesterol – high-density lipoprotein cholesterol. N – number of control measurements *per* QC event. MA – moving average.		

## Discussion

In this study we have shown that even in a laboratory with a small daily volume of testing, it is possible to make a risk-based QC plan that combines traditional QC and MA procedures. Namely, in papers about the use of run size calculators and earlier run size nomograms, authors discuss the need for this type of control in large laboratories (*9*, *20*, *25*). Similar goes for papers dealing with methods of implementing PBRTQC procedures (*4*, *26*). However, it is clear that laboratories with a small daily number of tests, such as our case, also need to minimize the risk of issuing an incorrect result.

One of the major and well-known challenges in making a QC plan in a medical laboratory occurs when several different tests, which do not all have the same performance, *i.e.*, the same sigma-metrics, are run on the same analyser (*9*). In order to make our QC plan as simple as possible for implementation in routine work, we decided on the approach of unifying the strategy, including as few different rules as possible (*20*). Therefore, only one start-up and one monitoring SQC rule were selected for all examined tests.

Regarding the implementation of PBRTQC into a traditional SQC plan, we agree with other authors that, though alone it is not enough, PBRTQC certainly deserves a place in routine laboratory practice, in addition to traditional control (*4*, *12*, *14*). Both traditional QC and PBRTQC have their advantages but also their disadvantages. Therefore, it is most rational to use them to complement each other (*3*, *4*, *13*). In an attempt to combine the best of these two control concepts, we presented in this study the potential use of MA control procedures in the case of tests with different sigma metric values. For tests with patient-risk sigma values higher than 5 (albumin and cholesterol) and, especially, higher than 6 (AST, HDL-cholesterol and potassium), we replaced the monitoring SQC procedures with MA procedures. The rational explanation for this substitution is based on 2 factors: (*1*) the small daily number of tests the laboratory performs relative to the run size between two control measurements allowed by the start-up rule and (*2*) the ability of optimized MA procedures to detect critical-size bias within the daily number of tests. Namely, for all 5 of these tests, the run size calculated for the start-up control rule is 1000 tests, which is 6.6 to 33.3 times more than the maximum daily number for each of these tests. At the same time, for albumin, AST, HDL-cholesterol and potassium, the optimized MA procedures will detect critical-size bias in one-fifth to four-fifths of the daily number of results, making them a reliable replacement for the SQC monitoring procedure. We included cholesterol in this group of tests, although the performance of its MA procedure is not as good as for the previous 4 analytes. We found justification for this in the fact that this test has a high sigma value. At the same time, the median number of results an optimized MA procedure requires to detect positive critical bias is between one-third and one-half of the daily number of tests. In contrast, the number of results needed to detect negative critical bias is close to the maximum daily number of tests. At the other end of the sigma scale, in tests with low or marginal performance, such as, in our case, sodium (sigma < 4) or calcium and total proteins (sigma just above 4), MA procedures also have their place, but as a supplement to the defined monitoring SQC procedures. Namely, the run size between two QC events is incomparably smaller than in tests with sigma > 4.5, and monitoring SQC procedures is necessary. But given the good performance of MA procedures (*i.e.*, the ability to detect clinically significant bias), we believe that MA provides additional security between two SQC events. In case a bias occurs in the analytical system, it will be signalled by an MA alarm, allowing us to perform the SQC without waiting for the interval provided by the plan to expire and thus to confirm or deny the existence of a problem requiring corrective action. The ability of the sodium MA procedure to detect clinically significant bias in one-third of the daily number of results prompted us to select the same SQC monitoring procedure for all tests that were to be performed, despite the calculated run size for sodium being discretely smaller than the maximum daily number of tests.

When it comes to tests from the medium sigma metrics group, we were more careful than with high sigma metrics tests, even though the run size covered by the start-up rule is 1000 tests, *i.e.*, many times greater than their daily number. Based on the characteristics of the MA procedure, we acted differently with chloride and creatinine. Since the MA procedure for chloride reveals a clinically significant bias in one-third of the daily number of tests, we classified it into tests in which MA is a replacement for the SQC monitor. In the case of creatinine, we decided to keep the monitoring SQC procedure because the existing MA procedure does not guarantee the detection of a positive critical bias within the daily test production.

Regardless of the value of the test’s sigma metrics, it should be noted that PBRTQC has the ability to detect both preanalytical and analytical errors; hence, adding this type of procedure to the QC plan can yield multiple benefits (*12*, *14*).

When developing a multistage QC plan, it should be kept in mind that increasing the frequency of QC inevitably increases the laboratory costs. This is particularly problematic for small laboratories where the percentage of controls in the total daily number of tests may become an unacceptable cost, even in the case of tests with good sigma metrics, unlike in laboratories with large testing volumes (*6*). In this regard, we believe it is an important finding of this paper that the inclusion of MA procedures in a risk-based QC plan contributed to reducing QC costs. For 5 out of 10 examined tests, this concept enabled replacing a monitoring SQC procedure with an optimized MA procedure. Other authors have already shown that the use of PBRTQC brings significant savings in both money and time (*27*).

One of the key parameters for calculating sigma metric and thus the run size between two QC measurements is the quality requirement of a specific test, expressed as TEa (*20*). When discussing the issue of data sources for TEa, there is no consensus in the current literature (*28*, *29*). In a way, it is left to individual laboratories to choose a performance specification according to their own practical needs. Therefore, we opted for CLIA data because we have used it before to calculate sigma metrics and because we find those performances achievable. At the same time, we are, of course, aware of the existence of other and far stricter performance specifications, including those based on biological variation (*13*, *28*, *30*). However, we know from the already published studies that MA procedures are inferior in detecting TEa based on biological variation, and our goal was to include PBRTQC in the QC plan (*13*, *23*). In the next period, we will certainly work on improving the sigma metrics of our tests: reducing CV and bias could potentially ensure the application of more stringent TEa.

Regarding the limitations of our study, we have only developed a risk-based QC plan for 10 clinical chemistry analytes but it should be applied to all tests performed by our laboratory. Also, it would be necessary to examine in routine practice how often the MA procedure will detect a problem before the scheduled monitoring SQC procedure.

In conclusion, we can say that the study has shown it is possible to make a risk-based QC plan even in a laboratory with a small daily volume of testing, and MA procedures deserve their place in this plan.
